# Universal light-guiding geometry for on-chip resonators having extremely high Q-factor

**DOI:** 10.1038/s41467-020-19799-2

**Published:** 2020-11-23

**Authors:** Dae-Gon Kim, Sangyoon Han, Joonhyuk Hwang, In Hwan Do, Dongin Jeong, Ji-Hun Lim, Yong-Hoon Lee, Muhan Choi, Yong-Hee Lee, Duk-Yong Choi, Hansuek Lee

**Affiliations:** 1 Department of Physics, Korea Advanced Institute of Science and Technology (KAIST), Daejeon, 34141 Republic of Korea; 2 Graduate School of Nanoscience and Technology, Korea Advanced Institute of Science and Technology (KAIST), Daejeon, 34141 Republic of Korea; 3 Department of Robotics Engineering, Daegu Gyeongbuk Institute of Science and Technology, Daegu, 42988 Republic of Korea; 4 School of Electronics Engineering, Kyungpook National University, Daegu, 41566 Republic of Korea; 5 Laser Physics Centre, Research School of Physics, Australian National University, Canberra, ACT 2601 Australia

**Keywords:** Optical materials and structures, Microresonators

## Abstract

By providing an effective way to leverage nonlinear phenomena in integrated devices, high-Q optical resonators have led to recent advances in on-chip photonics. However, developing fabrication processes to shape any new material into a resonator with extremely smooth surfaces on a chip has been an exceptionally challenging task. Here, we describe a universal method to implement ultra-high-Q resonators with any new material having desirable properties that can be deposited by physical vapor deposition. Using this method light-guiding cores with surface roughness on the molecular-scale are created automatically on pre-patterned substrates. Its efficacy has been verified using As_2_S_3_, a chalcogenide glass that has high-nonlinearity. The Q-factor of the As_2_S_3_ resonator so-developed approached the propagation loss record achieved in chalcogenide fibers which were limited by material losses. Owing to the boosted Q-factor, lasing by stimulated Brillouin scattering has been demonstrated with 100 times lower threshold power than the previous record.

## Abstract (web summary)

High-Q resonators are used to enhance nonlinear phenomena in photonics, but developing a fabrication process to shape a new material into a resonator is challenging and costly. Here, the authors present a method using vapour deposition on pre-patterned substrates to fabricate resonators with any new material.

## Introduction

High-*Q* optical resonators, which have long been used for scientific research into phenomena such as cavity quantum electrodynamics^1^ and optomechanics^2^, have become a valuable tool for on-chip photonics with huge practical impact. As a result of efforts over the past decade, on-chip resonators can now be fabricated with extreme *Q*-factors^3–6^. Their geometry can be precisely designed and shaped to control properties such as free spectral range (FSR) and dispersion to satisfy the requirements for efficient nonlinear processes^3,7,8^. These advances have allowed on-chip photonics to exploit phenomena from nonlinear optics while keeping its own virtues of miniaturization, mass productivity, and integration with other active and passive components. Optical frequency combs, which have revolutionized ultrafast science and metrology, can now be implemented on a chip using the Kerr nonlinearity^4,5^. Similarly, core attributes such as of ultra-narrow linewidth lasers^9^ and highly sensitive optical gyroscopes^10^ have been demonstrated using chip-scale devices that employ stimulated Brillouin scattering (SBS).

So far, these demonstrations have relied almost entirely on three materials, Si, SiO_2_, and Si_3_N_4_, to create photonic chips incorporating nonlinear high-*Q* resonators. There is considerable motivation to explore alternative materials such as those that have better nonlinearity that would lead to a decrease in the pump power to the level compatible with portable devices^11–14^. Materials with extended transmission windows would also lead to devices that could operate in the mid-infrared (mid-IR)^11^. As a result, a lot of attention is being paid to develop on-chip high-*Q* resonators from other promising materials.

For on-chip high-*Q* resonators, the most critical issue is to develop fabrication processes customized for the particular material that produces extremely smooth surfaces since the *Q*-factor is often limited by surface scattering loss. In the case of silica, the processes have been refined over a decade, and unfortunately similar efforts may be needed to understand and overcome the limitations introduced during fabrication with each new material. This impedes the further development of the technology.

To address this issue, we propose a universal approach by which high-*Q* resonators, or more generally light-guiding structures with extremely low loss, can be defined on a chip from a wide range of materials without the need to develop a specific etching process for that material. This method that is inspired by the concept of transformation optics is demonstrated by realizing the on-chip As_2_S_3_ resonator with a *Q*-factor of 1.44 × 10^7^, which approximates the performance of the chalcogenide fibers. Furthermore, it is confirmed that the enhanced *Q*-factor enables lasing by stimulated Brillouin process with a threshold power of 0.54 mW, which is 100 times lower than the previous record. In addition, to efficiently access the modes of resonators made of any materials, a new flip–chip coupling scheme has been developed with a coupling ideality of 0.92.

## Results

### Implementation of universal low-loss waveguide

**Fig. 1:**
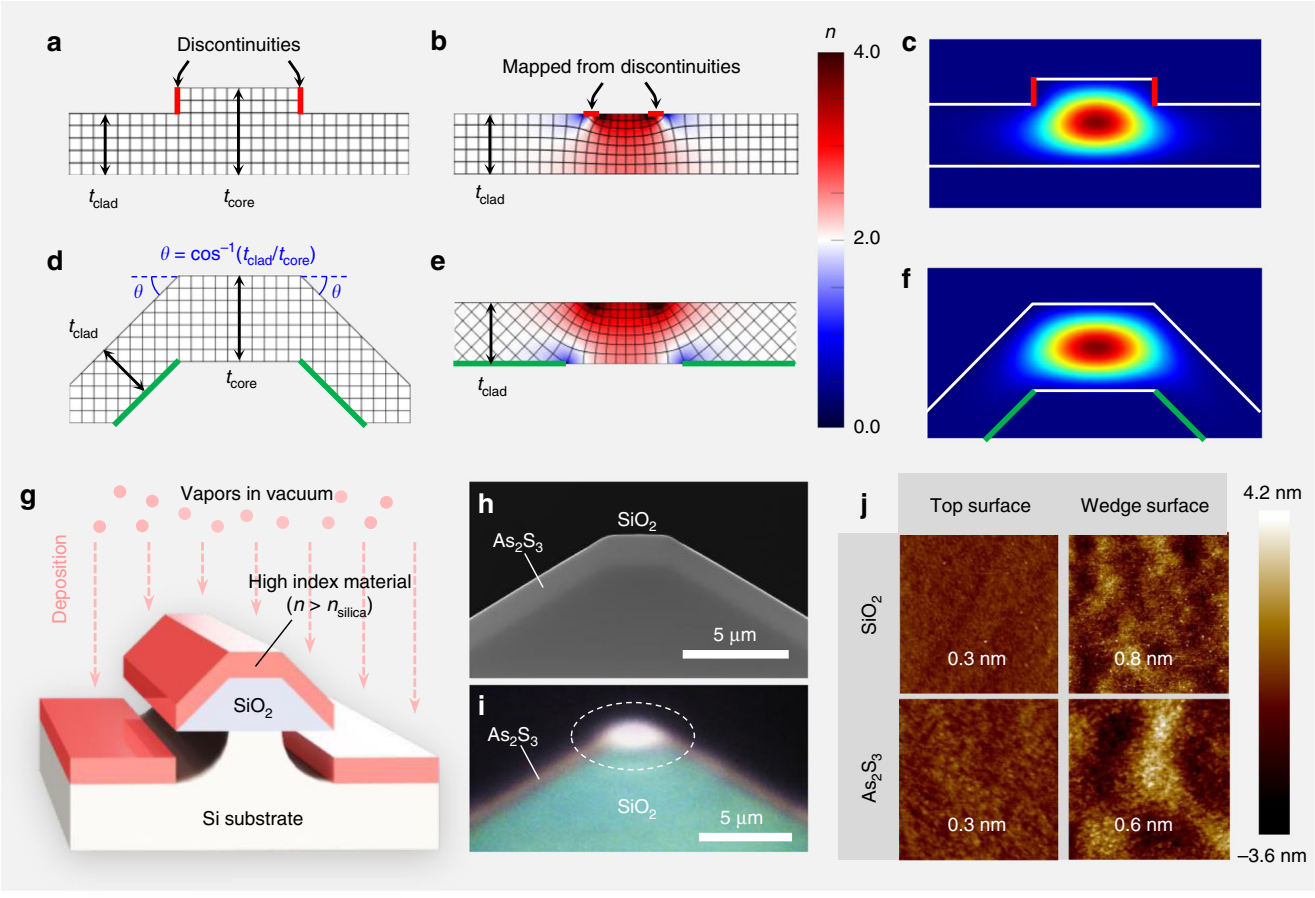
Design concept of a trapezoidal waveguide and its implementation. **a**, **d** A cross-section of a typical rib waveguide and the proposed waveguide without the discontinuities, respectively. The refractive index of the material is set to 2 (*n* = 2) without loss of generality. **b**, **e** A refractive index profile of the flat waveguides conformally mapped from the rib and the proposed waveguide, respectively. **c, f** A simulated optical mode profile of the rib and the proposed waveguides made of As_2_S_3_ (*n* = 2.43), respectively. **g** Implementation of the proposed waveguide. By depositing the core material on the SiO_2_ platform structure, the waveguide having continuous junctions can be formed. **h** The cleaved cross-section of the fabricated waveguide observed by scanning electron microscope. **i** An optical microscope image of the transmitted mode at the output facet. An IR camera image is superimposed with a visible camera image to clearly display the optical mode and waveguide structure. **j** AFM images showing surface roughness of As_2_S_3_ and SiO_2_ layers over 2 × 2 μm^2^ area on the top and wedge surfaces of the waveguide. The number shows the rms value.

Normally etching or subtractive processes are considered to be indispensable for waveguide fabrication because of the need for discontinuities in the waveguide geometry. Transformation optics^15^ provides insight into the nature of the discontinuity. Forming a light-guiding structure can be generally understood as the practical implementation of a core, with a higher refractive index than the surrounding medium. For the most common case where only a single material is used, the core is defined by controlling the thickness distribution as a rib waveguide shown in Fig. 1a. The refractive index profile of the equivalent flat waveguide in Fig. 1b, which is quasi-conformally transformed from the rib, clearly shows a higher index in the region corresponding to the thickest part of the original structure. The detailed methodology for the quasi-conformal mapping is explained in “Methods.” The mapping is performed to result in an equivalent structure having a uniform thickness while keeping the geometry of outer thinner parts, namely, the cladding, unchanged. It thus converts the thickness variation to an index distribution. It is noteworthy that, in conventional approaches, a discontinuity or sudden change in the film geometry necessarily occurs around the junction between the thick core and thin cladding as marked by the red lines in Fig. 1a. Therefore, to fabricate such discontinuities, a direct etching process, or an indirect process such as lift-off, is inevitable, and these can cause rough surfaces. Moreover, these rough surfaces strongly interact with the optical modes since they are mapped near the highest index region where most of the field is confined as shown in Fig. 1c.

To resolve this problem, we introduce a new light-guiding structure without these discontinuities as shown in Fig. 1d. To continuously connect the claddings having a thickness of *t*_clad_ to the core having a thickness of *t*_core_, the claddings are rotated by angle *θ* that satisfies the condition cos*θ* = *t*_clad_/*t*_core_ (−90° < *θ* < 90°). The transformed geometry in Fig. 1e clearly shows the formation of a high index region around the top flat section. This waveguide that has continuous junctions can be realized simply by depositing the core material on the bottom platform structure made of SiO_2_ without any need to etch the core. As shown in Fig. 1g, the cross-section of the platform structure is a trapezoid having both side slopes corresponding to the characteristic profile formed by wet etching^3^. Directional physical vapor deposition of the core material results in a uniform film thickness of *t*_core_ along the deposition direction over the whole platform. Therefore, the film thickness on the side slope along the normal direction from the material interface decreases to *t*_core_ cos*θ*, which satisfies the continuous junction condition for arbitrary values of *t*_core_ and *θ*.

This design of a trapezoidal waveguide involves an intrinsic immunity against any surface roughness of the platform structure. In contrast to the rib waveguide, the etched surfaces of the silica platform marked by the green lines in Fig. 1d are well separated from the high index region in the transformed geometry. Therefore, the optical modes are mainly guided by the surfaces that have molecular roughness defined by film deposition and the unetched horizontal oxide interface. Moreover, it is well known that the roughness of the wet-etched silica surface can be <1 nm as demonstrated for ultra-high-*Q* wedge resonators^3^.

This design is applicable to any material with a refractive index higher than SiO_2_ that can be directionally deposited onto a patterned wafer. Here we experimentally verify the process using a chalcogenide glass. Chalcogenide glasses have attracted great attention due to their high nonlinearity as well as high transparency in the mid-IR^11,16,17^. However, their inherent irregular nanostructure related to variations of stoichiometry and bond structures tends to result in a characteristic nanoscale roughness when etched due to nonuniformity in the local etch rate^18^. Over the past decade, there have been pioneering works to address this issue by optimizing direct etching^19,20^ or introducing indirect pattering methods^21,22^. Nevertheless, the loss of on-chip chalcogenide devices has lagged far behind that of chalcogenide optical fiber^17^, micro-sphere^23,24^, or theoretical estimations^25^.

The cross-sectional image of the waveguide fabricated with As_2_S_3_, a common chalcogenide glass, is shown in Fig. 1h. The film was uniformly deposited onto the SiO_2_ platform by thermal evaporation with a large mean free path (around 500 m at 10^−7^ torr). The surface roughness of the SiO_2_ platform and As_2_S_3_ film was <1 nm as confirmed by atomic force microscopic (AFM) image in Fig. 1j. By uniform deposition, As_2_S_3_ film showed less roughness than the original roughness of the SiO_2_ etched surface. The fabrication procedure and details are described in “Methods.”

The confinement of the optical mode in the top flat region was experimentally verified by observing the transmitted mode profile from the cleaved output facet of a straight waveguide with a near-IR camera as shown in Fig. 1i. The mode profile at 1550 nm corresponded accurately to the intensity distribution of the fundamental transverse electric (TE) mode in Fig. 1f simulated by Lumerical Mode Solutions. The edge corners on the top outer surface of the deposited film can be rounded during practical deposition (see Fig. 1h). It is confirmed that these rounded corners barely affect the shape and properties of the optical modes, as described in Supplementary Note 1. The coupling loss for end-fire coupling with a lensed fiber was 3.5 dB, which is well agreed with the coupling loss of 3.4 dB numerically calculated from the mode overlap.

### Characterization with flip–chip coupling scheme

Although on-chip ring resonators can be easily implemented with trapezoidal waveguides, an appropriate coupling scheme is required to efficiently excite the modes in the resonators. Efficient coupling to chalcogenide resonators has been a critical issue because of its relatively higher refractive index that cannot be matched to a SiO_2_ tapered fiber, the most common coupling method^26,27^. Although there have been considerable efforts to fabricate tapered fibers from chalcogenide glass^28^, their fragility and the difficulty of the tapering process has inhibited widespread use of this technique.

**Fig. 2:**
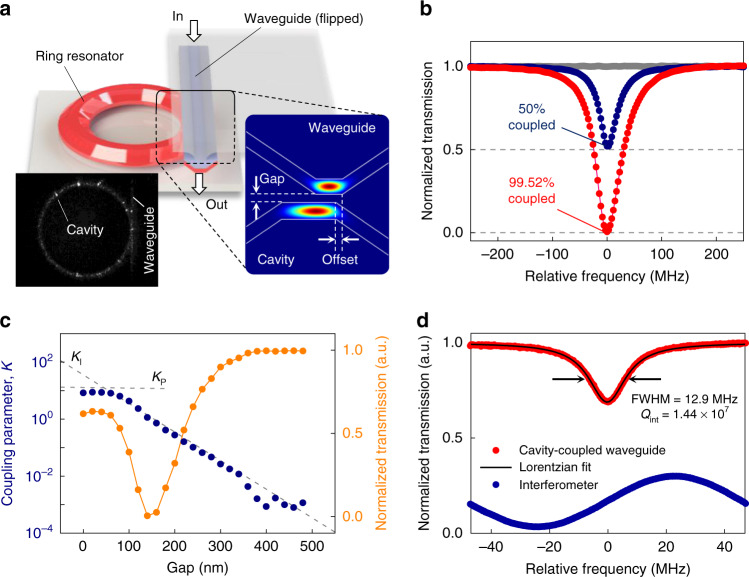
Optical coupling between the trapezoidal waveguide and the ring resonator. **a** Schematic of the flip–chip coupling scheme. Optical modes in the waveguide and the resonator are evanescently coupled through their top surfaces. Coupling strength is controlled by adjusting the gap and the offset (inset, right). The left inset taken by IR camera shows the scattered light trace from the coupled chips. **b** Transmission spectra with different coupling strengths. **c** The gap versus coupling parameter *K* (navy curve) converted from the normalized transmission *T* (orange curve). The gap is obtained by monitoring the position of the piezo stage. When waveguide transmission was significantly dropped, the gap is determined to 0 nm. **d** Transmission spectrum for measuring *Q*-factor of the trapezoidal resonator (red curve) and its Lorentzian fitting (black line). Intrinsic *Q*-factor of 14.4 million is obtained from the spectrum. The navy curve is the calibration spectrum from a fiber Mach–Zehnder interferometer.

As a general solution for coupling resonators made of any materials, a new flip–chip coupling scheme has been developed as shown in Fig. 2a. Here an on-chip bus waveguide is flipped over and coupled to the resonator on a second chip. Since both guiding structures have a similar trapezoidal cross-section, the effective refractive indices of their modes match well. The image of light scattered by the mode in the coupled chips, which was observed with the IR camera through the flipped-over chip, is shown in the left inset of Fig. 2a. The coupling strength can be adjusted as needed by controlling the gap and offset (Fig. 2a right inset) with a piezo stage. As confirmed in Fig. 2b, the resonant mode in under-coupled and critical-coupled conditions clearly fits with Lorentzian function without a vestigial Fano resonance, which is typically observed when using a multimodal bus waveguide. The details of the design of the single-mode bus waveguide and the suppression of the Fano resonance can be found in Supplementary Notes 2–5.

For further investigation of the performance of this coupling junction, the coupling parameter *K* and the ideality *I* have been calculated from the transmission on resonance measured by varying the gap distance between the waveguide and the resonator^29,30^. Since the coupling parameter can be adjusted ≥1 as shown in Fig. 2c, it is confirmed that the over-coupling condition beyond the critical coupling is achievable. The coupling performance is quantified by the ideality of the junction—that is the degree to which the junction between a bus waveguide and resonator behaves as a true single-mode coupler. As described in detail in Supplementary Note 6, it was confirmed that the ideality value is 0.923 in the critical coupling condition. Here zero ideality means no coupling into the desired mode and unity stands for the perfect single-mode coupler. Therefore, the measured ideality shows that the coupling scheme allows selective and efficient access to the targeted mode without significant power loss or additional complexity of multimode interactions.

The *Q*-factor, the most important feature, was measured to evaluate the performance of the resonator. The dimensions of the resonator were 1.3 µm for the As_2_S_3_ film thickness, 10 µm top width, 30° slope angle, and 4.94 mm diameter. By scanning the laser frequency, the full width half maximum (FWHM) of the resonance of the TE mode was measured and calibrated by a sinusoidal reference signal at 100.63 MHz from a fiber Mach–Zehnder interferometer as shown in Fig. 2d. The *Q*-factor obtained from the measured FWHM of 12.9 MHz was 1.44 × 10^7^, which is >10 times larger than the previous record for the on-chip resonators made of chalcogenide glass. It should be emphasized that the waveguide loss of 2.77 dB m^−1^ converted from the measured *Q*-factor approaches 0.9 dB m^−1^, the best record of the loss of chalcogenide glass fiber at conventional telecom wavelengths^17^. Using a volume current method and an autocorrelation function for surface roughness^31,32^, the scattering loss of the trapezoidal waveguide is calculated to be 1.62 dB m^−1^. This implies that the scattering loss induced by the trapezoidal waveguide is well suppressed to the level of the material loss of chalcogenide glass, which is dominated by material absorption and Rayleigh scattering by nanoscale refractive index fluctuations. The trapezoidal waveguide can also support transverse magnetic (TM) modes. The polarization-dependent loss performance is discussed in detail in Supplementary Note 7.

### Demonstration of Brillouin laser

**Fig. 3:**
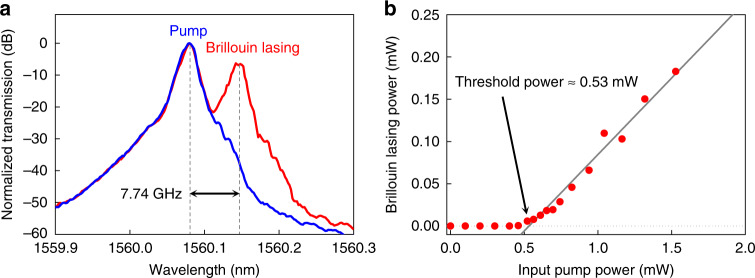
Brillouin laser with a sub-mW threshold power using a high-*Q* resonator. **a** Optical spectra obtained by an optical spectrum analyzer with pump powers below threshold (blue line) and above threshold (red line). The Brillouin shift of 7.74 GHz was measured by an electrical spectrum analyzer. **b** Input pump power versus Brillouin lasing power. A lasing threshold power of 0.53 mW is obtained by a linear fitting of the data (gray line).

From the possible applications exploiting the large optical nonlinearity of chalcogenide glass, we demonstrate here on-chip lasing based on the SBS process. The dimensions of the resonator were the same used for the *Q*-measurement. The FSR of the TE fundamental mode of these resonators was 7.74 GHz and was designed to correspond to the Brillouin phonon frequency in As_2_S_3_ at 1560 nm. Characterization of the Brillouin lasing signal was performed using the measurement set-up similar to previous studies^10,33^. Figure 3a shows the measured optical spectrum of the back-reflected pump and the Brillouin lasing signal (first Stokes wave). In this figure, the pump intensity is higher than the Brillouin lasing signal because around 17.5% of the pump was back-reflected at the input facet of the coupled waveguide due to the large difference of refractive index between the air and chalcogenide glass.

A particularly low lasing threshold was achieved because of the enhanced *Q*-factor and large Brillouin gain coefficient of As_2_S_3_ (tens of times larger than values for SiO_2_ and Si_3_N_4_). The dependence of the lasing signal power on the input pump power was measured while the pump laser was locked to the resonant mode and is plotted in Fig. 3b. It shows a typical lasing curve with a slope efficiency of 18%. The lasing threshold was 530 µW, which is around 100 times lower than the previous record for on-chip Brillouin lasers made of chalcogenide glass^34^ and 25 times lower than values reported for silicon nitride^9^. It is worth noting that the threshold power of not only the Brillouin lasing but also the other nonlinear processes such as Raman lasing and four wave mixing is inverse-quadratically related to the *Q*-factor^35^.

## Discussion

In summary, we have presented the universal approach to defining the light-guiding structure with extremely smooth surfaces on a chip without etching the core as well as a flip–chip coupling scheme to evanescently couple to high-*Q* resonators with high coupling ideality. By applying this scheme to chalcogenide glass, the threshold of the on-chip Brillouin laser has been reduced by about 100 times compared with values for previous chalcogenide devices because of the enhanced *Q*-factor. The waveguide losses are close to the state of the art achieved in optical fibers made of the same material. This universal method can be applied to bring the other materials with favorable properties to on-chip photonics while keeping the loss performance comparable to that of bulk material.

Additional features proving the potential of this approach are briefly described as follows. It has been numerically demonstrated that dispersion can be geometrically controlled by adjusting the structure of the trapezoidal waveguides. In Supplementary Note 8, the dispersion engineering for the trapezoidal waveguides is described in detail. Furthermore, since it is easy to stack multilayer films with thickness controllable on the nanometer scale, precise dispersion control using different combinations of material is also possible. For mid-IR applications, material absorption due to the SiO_2_ platform can be completely eliminated by depositing a mid-IR transmitting bottom cladding layer before core deposition. The detailed discussion can be found in Supplementary Note 9.

Although the feasibility of the proposed method is experimentally verified with a material having a higher refractive index than silica and physical vapor deposition providing good directionality, the universality of this method is further discussed in Supplementary Note. In Supplementary Notes 10 and 11, we show examples of the modified structures to employ conformal deposition processes and materials having a lower refractive index than silica, respectively.

## Methods

### Obtaining quasi-conformal mapping

We have found the quasi-conformal mapping that maps the boundaries of the virtual domain (trapezoidal or rib shape) to the specified boundaries in the physical domain (flat rectangular shape). Under a general coordinate transformation from virtual space (*η* = *u* + *iv*) to physical space (*ζ* = *x* + *iy*), the form of Maxwell’s equations is preserved with the following constitutive parameters

$$\varepsilon^{\prime} = \frac{{{\Lambda}\varepsilon {\Lambda}^{\mathrm{T}}}}{{\left| {\det {\Lambda}} \right|}},\quad \mu^{\prime} = \frac{{{\Lambda}\mu {\Lambda}^{\mathrm{T}}}}{{\left| {\det {\Lambda}} \right|}},$$

where ${\Lambda} = \frac{{\partial \left( {x,y} \right)}}{{\partial \left( {u,v} \right)}}$ is the Jacobian matrix.

Especially for conformal mapping *ζ* = *f*(*η*), the Cauchy–Riemann equations highly simplify the above tensorial parameters to the following refractive index profile $n{\prime} = n\left| {\frac{{{\mathrm{d}}\zeta }}{{{\mathrm{d}}\eta }}} \right|^{ - 1}$ for TE- or TM-polarized electromagnetic waves^15^. In addition, by the Riemann mapping theorem, there exists a unique conformal mapping that maps a simply connected domain to an arbitrary simply connected domain. Therefore, there exists a conformal mapping that maps trapezoidal and rib shape to flat rectangular shape^36^.

The conformal mapping can be numerically found by calculating an extremal value of the Winslow functional with a fully sliding boundary condition.

$${\Phi} = {\int\!\!\!\!\!\int} {\frac{{{\mathrm{Tr}}\left( g \right)}}{{\sqrt {\det \left( g \right)} }}} D\left[ {u,v} \right],$$

where *g* is a metric tensor (ΛΛ^T^). Alternatively, to obtain an extremal value of that, one can solve the following Laplace equations

$$\frac{{\partial ^2x}}{{\partial u^2}} + \frac{{\partial ^2x}}{{\partial v^2}} = 0,\;\frac{{\partial ^2y}}{{\partial u^2}} + \frac{{\partial ^2y}}{{\partial v^2}} = 0,$$

which are the Euler–Lagrange equations of the Winslow functional^37^.

In principle, we can find the conformal mapping by solving Laplace equations with a fully sliding boundary condition. However, for the practical purpose, we obtained quasi-conformal mapping with partially sliding boundary condition on the upper and lower boundary of the waveguide while the boundary of left and right sides are fixed ($\left. y \right|_{{\mathrm{upper,}}\,{\mathrm{lower}}\,{\mathrm{boundary}}}{\mathrm{ = const,}}\,\left. {\frac{{\partial x}}{{\partial n}}} \right|_{{\mathrm{upper,}}\,{\mathrm{lower}}\,{\mathrm{boundary}}} = 0,$ where $\frac{\partial }{{\partial n}}$ is normal derivative at the boundary of interest in the virtual space). As a result, we can find quasi-conformal mapping but the anisotropic factor is almost 1 (≈1.001), which implies that it is almost conformal.

### Formation of SiO_2_ trapezoidal platform

**Fig. 4:**
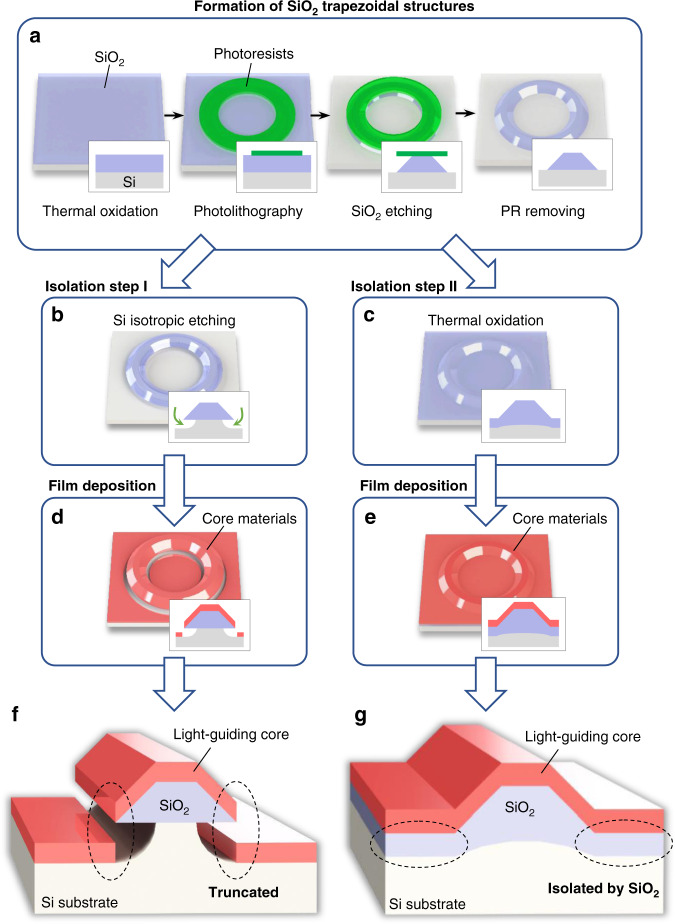
Fabrication procedure to realize the high-*Q* resonator (or the trapezoidal waveguide) on a chip. **a** Sequence to form SiO_2_ trapezoidal structures. **b** Isotropic XeF_2_ etching of a Si substrate to isolate an optical field from the substrate. **c** Additional thermal oxidation as an alternative to the isolation step. **d** Film deposition of core materials on the XeF_2_ etched structure. **e** Film deposition of core materials on the additionally oxidized structure. **f** The XeF_2_-etched high-*Q* resonator in which the film of the core material is truncated at the end of the wedge structure. **g** The additionally oxidized high-*Q* resonator in which the film of the core material is isolated from Si substrate by the SiO_2_ sublayer.

Figure 4 shows the approach for forming SiO_2_ trapezoidal platform in detail. First, a layer of SiO_2_ was thermally grown with a thickness of 8 µm on a prime-grade (100) Si wafer in a furnace system (Tytan Mini 1800, TYSTAR). The SiO_2_ layer was treated with hexamethyldisilazane (HMDS) vapor prime process to modify the surface adhesion property. To define the SiO_2_ trapezoidal platform, we then patterned photoresist on the SiO_2_ layer with a mask aligner lithography system (MA6 Mask Aligner, Karl Suss). The patterned photoresist was used as an etch mask for the following wet-etching process. The wet etching of SiO_2_ layer was done with buffered hydrofluoric acid solution. The slope angle of the platform could be controlled in the range from 5° to 60° according to the adhesion property between the photoresist and the SiO_2_ layer, which is mainly controlled with the HMDS treatment step. In addition, by repeating the steps through the photolithography and wet etching, the slope angle could reach larger values than the normal range attained by a single process^7^. The details for realizing the smooth etched surface with the desired slope angle are described in the previous work^3^. After wet etching, a cleaning process with organic solvents was performed to remove the photoresist.

### Isolation from Si substrate

Once the SiO_2_ trapezoidal platform had been formed, the Si substrate under the platform was isotropically etched with XeF_2_ gas (XeF_2_ etching system, Teraleader Co. Ltd.) as shown in Fig. 4b. By having this step, the optical field at the trapezoidal waveguide was perfectly isolated from the Si substrate to prevent leakage to the substrate. Alternatively, the optical field can be isolated from the substrate by growing a thermal oxide film on the Si substrate exposed by wet etching (Fig. 4c).

### Film deposition

Once the isolation step was completed, a material deposition step followed (Fig. 4f, g). The material we use for the light-guiding core in this paper was As_2_S_3_ (AMTIR-6, Amorphous Materials Inc.), and the thickness was 1.3 µm. The material was deposited with a thermal evaporation process, and the deposition direction was normal to the wafer substrate. Crystallite formation on chalcogenide films, in particular in Arsenic-based ones, is a common issue. After the deposition, a 2-nm-thick Al_2_O_3_ layer was formed on the surface by atomic layer deposition for surface passivation. A two-step annealing process (thermal and light annealing) reported in the previous work was performed to bring the As_2_S_3_ film close to the bulk state, which is much more resilient to such crystallization^38^. To estimate the surface roughness of the waveguide, we analyzed the topography of the waveguide surfaces using AFM. On the bare SiO_2_ structure, the rms roughness on the top and the wedge was 0.3 and 0.8 nm, respectively. Whereas on the as-deposited As_2_S_3_ surface, the roughness on the top and the wedge was 0.3 and 0.6 nm, respectively. The other fabrication and measurement information for the As_2_S_3_ resonators can be also found in our conference proceedings^39^.

### Calculating coupling ideality, *I*

Details for calculating coupling parameters and ideality can be found in the previous studies^29,30^ but are summarized here for the convenience of the reader. The coupling parameter *K* can be calculated from the transmission of the waveguide *T* (coupled to the resonator at resonant wavelength) using the following equation $K = \left( {1 \pm \sqrt T } \right)/\left( {1 \mp \sqrt T } \right)$ (upper signs for the over-coupled regime and lower signs for the under-coupled regime). *K* can be decomposed into an intrinsic contribution *K*_I_ and a parasitic contribution *K*_P_, which stands for the coupling of a resonator mode to a fundamental mode and the other various parasitic components including higher-order modes, respectively, so that 1/*K* = 1/*K*_I_ + 1/*K*_P_. *K*_I_ and *K*_P_ can be found by asymptotically fitting gap versus *K* graph (in logarithmic scale) with two lines (Fig. 2c). The line with a smaller slope indicates *K*_I_ and the line with a larger slope indicates *K*_P_. Coupling ideality *I* can be calculated from *K*_P_ by using the following equation $I = 1/\left( {1 + K_{\mathrm{P}}^{ - 1}} \right)$. The parasitic coupling parameter, which is clearly revealed in the gap <50 nm in the figure, gives the ideality value of 0.923 in the critical coupling condition. More details can be found in Supplementary Note 5.

## Supplementary information

Supplementary Information

## Data Availability

The data that support the findings of this study are available from the corresponding author upon reasonable request.

## Code availability

The codes that support the findings of this study are available from the corresponding author upon reasonable request.

## References

[1] Aoki T (2006). Observation of strong coupling between one atom and a monolithic microresonator. Nature.

[2] Kippenberg TJ, Vahala KJ (2008). Cavity optomechanics: back-action at the mesoscale. Science.

[3] Lee H (2012). Chemically etched ultrahigh-Q wedge-resonator on a silicon chip. Nat. Photonics.

[4] Spencer DT (2018). An optical-frequency synthesizer using integrated photonics. Nature.

[5] Stern B, Ji X, Okawachi Y, Gaeta AL, Lipson M (2018). Battery-operated integrated frequency comb generator. Nature.

[6] Xuan Y (2016). High-Q silicon nitride microresonators exhibiting low-power frequency comb initiation. Optica.

[7] Yang KY (2016). Broadband dispersion-engineered microresonator on a chip. Nat. Photonics.

[8] Kim S (2017). Dispersion engineering and frequency comb generation in thin silicon nitride concentric microresonators. Nat. Commun..

[9] Gundavarapu S (2019). Sub-hertz fundamental linewidth photonic integrated Brillouin laser. Nat. Photonics.

[10] Lai YH, Lu YK, Suh MG, Yuan Z, Vahala K (2019). Observation of the exceptional-point-enhanced Sagnac effect. Nature.

[11] Eggleton BJ, Luther-Davies B, Richardson K (2011). Chalcogenide photonics. Nat. Photonics.

[12] Wang C (2019). Monolithic lithium niobate photonic circuits for Kerr frequency comb generation and modulation. Nat. Commun..

[13] Pu M, Ottaviano L, Semenova E, Yvind K (2016). Efficient frequency comb generation in AlGaAs-on-insulator. Optica.

[14] Li L (2014). Integrated flexible chalcogenide glass photonic devices. Nat. Photonics.

[15] Xu L, Chen H (2015). Conformal transformation optics. Nat. Photonics.

[16] Petersen CR (2014). Mid-infrared supercontinuum covering the 1.4–13.3 μm molecular fingerprint region using ultra-high NA chalcogenide step-index fibre. Nat. Photonics.

[17] Kim WH (2016). Recent progress in chalcogenide fiber technology at NRL. J. Non-Cryst. Solids.

[18] Choi DY, Madden S, Rode A, Wang R, Luther-Davies B (2007). Nanoscale phase separation in ultrafast pulsed laser deposited arsenic trisulfide (As 2 S 3) films and its effect on plasma etching. J. Appl. Phys..

[19] Madden SJ (2007). Long, low loss etched As_2_S_3_ chalcogenide waveguides for all-optical signal regeneration. Opt. Express.

[20] Du Q (2016). Low-loss photonic device in Ge–Sb–S chalcogenide glass. Opt. Lett..

[21] Hu J (2007). Si-CMOS-compatible lift-off fabrication of low-loss planar chalcogenide waveguides. Opt. Express.

[22] Kang G (2017). High quality chalcogenide-silica hybrid wedge resonator. Opt. Express.

[23] Broaddus DH (2009). Silicon-waveguide-coupled high-Q chalcogenide microspheres. Opt. Express.

[24] Vanier F, Rochette M, Godbout N, Peter YA (2013). Raman lasing in As_2_S_3_ high-Q whispering gallery mode resonators. Opt. Lett..

[25] Dianov EM, Petrov MY, Plotnichenko VGE, Sysoev VK (1982). Estimate of the minimum optical losses in chalcogenide glasses. Sov. J. Quantum Electron..

[26] Knight JC, Cheung G, Jacques F, Birks TA (1997). Phase-matched excitation of whispering-gallery-mode resonances by a fiber taper. Opt. Lett..

[27] Cai M, Painter O, Vahala KJ (2000). Observation of critical coupling in a fiber taper to a silica-microsphere whispering-gallery mode system. Phys. Rev. Lett..

[28] Lecaplain C, Javerzac-Galy C, Gorodetsky ML, Kippenberg TJ (2016). Mid-infrared ultra-high-Q resonators based on fluoride crystalline materials. Nat. Commun..

[29] Spillane SM, Kippenberg TJ, Painter OJ, Vahala KJ (2003). Ideality in a fiber-taper-coupled microresonator system for application to cavity quantum electrodynamics. Phys. Rev. Lett..

[30] Pfeiffer MH, Liu J, Geiselmann M, Kippenberg TJ (2017). Coupling ideality of integrated planar high-Q microresonators. Phys. Rev. Appl..

[31] Johnson SG (2005). Roughness losses and volume-current methods in photonic-crystal waveguides. Appl. Phys. B.

[32] Kita DM, Michon J, Johnson SG, Hu J (2018). Are slot and sub-wavelength grating waveguides better than strip waveguides for sensing?. Optica.

[33] Eggleton BJ, Poulton CG, Pant R (2013). Inducing and harnessing stimulated Brillouin scattering in photonic integrated circuits. Adv. Opt. Photonics.

[34] Morrison B (2017). Compact Brillouin devices through hybrid integration on silicon. Optica.

[35] Boyd, R. W. *Nonlinear Optics* (Academic, 2019).

[36] Park SJ, Kim I, Cho J, Kim Y, Choi M (2019). Designing arbitrary-shaped whispering-gallery cavities based on transformation optics. Opt. Express.

[37] Chang Z, Zhou X, Hu J, Hu G (2010). Design method for quasi-isotropic transformation materials based on inverse Laplace’s equation with sliding boundaries. Opt. Express.

[38] Choi DY (2013). Photo-induced and thermal annealing of chalcogenide films for waveguide fabrication. Phys. Proc..

[39] Han, S. et al. On-chip Brillouin lasers based on 10 million-Q chalcogenide resonators without direct etch process. In *Laser Resonators, Microresonators, and Beam Control XXII* Vol. 11266, (eds Kudryashov, A. V., Paxton, A. H., Ilchenko, V. S. & Armani, A. M.) 112660 (SPIE, International Society for Optics and Photonics, 2020).

