# Supplementary material for: Universal light-guiding geometry for on-chip resonators having extremely high Q-factor
Source: Nat Commun. 2020 Nov 23;11:5933. doi: 10.1038/s41467-020-19799-2 (PMC7683556; doi:10.1038/s41467-020-19799-2)
Supplement: Supplementary file 1 — Supplementary Information [file 41467_2020_19799_MOESM1_ESM.pdf]

Supplementary Information - Universal light-guiding geometry for on-chip resonators having extremely high Q-factor

Kim *et al*.

Supplementary Note 1. PERTURBATION BY ROUNDED CORNERS

As shown in supplementary figure 1, to evaluate how the rounded corners affect mode shapes, the optical simulation is performed with a mode solver for the edged and the rounded corners, respectively. For the rounded corner, we used a contour captured from the SEM image of the fabricated devices. The mode profiles for the two waveguides are quite similar and have a power overlap of 0.99997. The differences of the effective refractive indices and group delay dispersion values for both modes were only 0.004% and 0.03%, respectively.

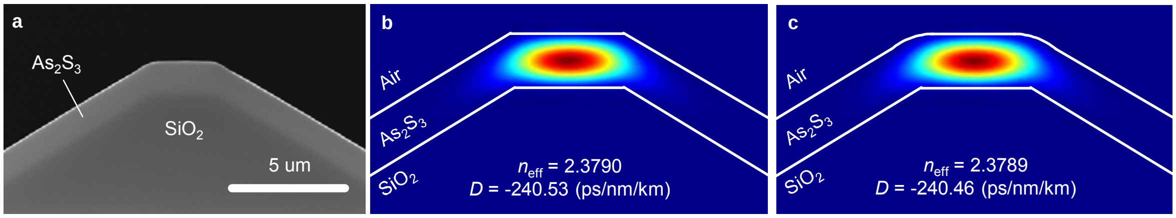

Supplementary Figure 1. How rounding corners change the optical properties of the fundamental TE mode. a The SEM image for a cleaved facet of the fabricated waveguide. **b,c** The $|E|^2$ profile of the edged and rounded waveguides with their refractive index and group delay dispersion.

Supplementary Note 2. SINGLE MODE CONDITION FOR TRAPEZOIDAL WAVEGUIDES

Supplementary figure 2 shows geometrical conditions for the proposed trapezoidal waveguides to support only one transverse electric mode at their top-flat regions. The number of modes

supported at the top-flat regions is calculated using Lumerical Mode Solutions. The thickness and refractive index of waveguide cores are respectively set to 1.3 μm and 2.437 (refractive index of As_2S_3 at 1550 nm wavelength), while the top width and the slope angle are varied. A line connecting dots in supplementary figure 2 indicates a boundary between single-mode (below the line) and multi-mode regions (above the line). The core thickness, the top width, and the slope angle of the bus waveguide used in the coupling experiments are 1.3 μm, 2.7 μm, and 31°, respectively. As marked in supplementary figure 2, the bus waveguide satisfies the single-mode condition.

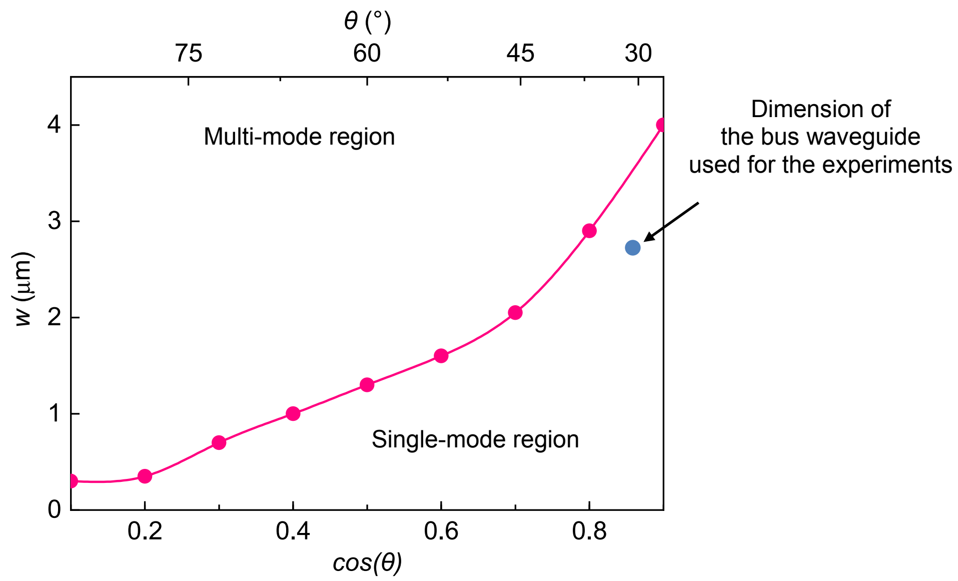

Supplementary Figure 2. Geometrical conditions for trapezoidal waveguides to be single-mode or multi-mode waveguides. The line connecting dots implies a boundary between single-mode (below the line) and multi-mode (above the line) regions. The thickness and refractive index of the waveguide core are set to 1.3 μm and 2.437 (refractive index of As_2S_3 at 1550 nm

wavelength), while top width and slope angle are varied along the axes. The blue dot indicates a geometry of the bus waveguide used in the coupling experiments of the main text.

Supplementary Note 3. FABRY-PÉROT RESONANCE OF WAVEGUIDES

Supplementary figure 3 shows measured transmission spectra of a trapezoidal waveguide coupled to a trapezoidal resonator near 1550 nm wavelength. Besides sharp resonance peaks from the cavity resonance, there is a periodic fluctuation of transmission spectra due to Fabry-Pérot resonance from the waveguide. The two facets of the waveguide act as partial reflecting mirrors and therefore the waveguide act as a Fabry-Pérot cavity. The measured free spectral range (FSR) of the Fabry-Pérot mode is 0.026 nm as marked in supplementary figure 3a, and it corresponds to the length of the waveguide which is 1.8 cm[1]. The Fabry-Pérot resonance can be suppressed by reducing the reflectance of the facets by having anti-reflection coating at the waveguide facets[2].

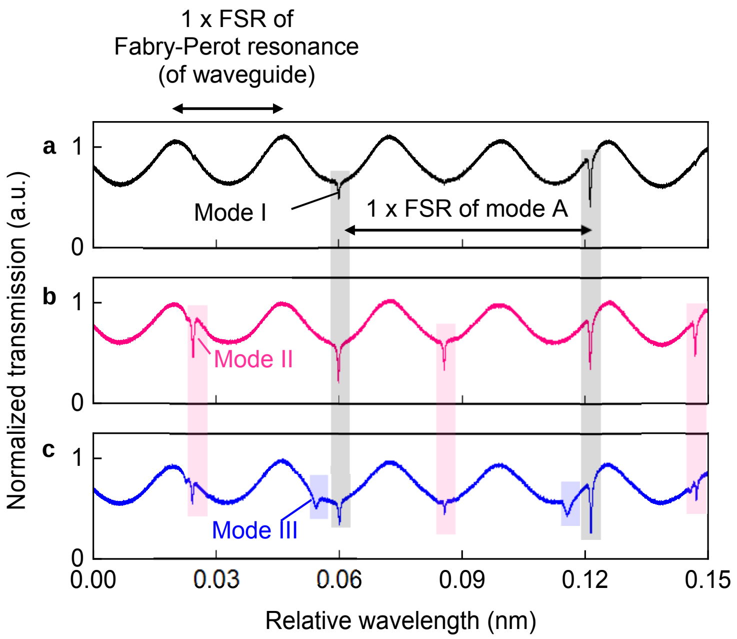

Supplementary Figure 3. Transmission spectra of the trapezoidal waveguide coupled to the trapezoidal resonator with different alignment offsets. a The Fabry-Perot resonance of the waveguide is marked on the graph. Only one mode family (Mode I) of the resonator is coupled to the waveguide. **b** By changing the lateral offset between the waveguide and the resonator, two mode families are accessible (Mode I and II). **c** With a further change in the offset, three mode families are accessible (Mode I, II, and III).

Supplementary Note 4. CONTROLLING THE NUMBER OF ACCESSIBLE MODES BY WAVEGUIDE-TO-RESONATOR ALIGNMENT

With the flip-chip coupling scheme we presented in this paper, one can change the number of modes coupled to the waveguide by changing the lateral offset between the waveguide and the resonator. In supplementary figure 3a, an only one mode family of the resonator is coupled. By

changing the lateral offset between the resonator and the waveguide, one can couple two mode families at the same time (supplementary figure 3b). With further adjustment of the lateral offset, one can couple three mode families at the same time (supplementary figure 3c).

Supplementary Note 5. SUPPRESSING FANO RESONANCE BY WAVEGUIDE DIMENSION CONTROL

When more than one mode is supported by a waveguide, the transmission spectrum of the waveguide coupled to a resonator exhibits Fano resonance[3], and it complicates the analysis of a waveguide-resonator system. Thus, one needs a single-mode waveguide to suppress the Fano resonance. By narrowing the top width of a trapezoidal waveguide, the waveguide can satisfy the single-mode condition as described in supplementary figure 4. We prepared two waveguides with the same slope angle (= 31°) and core thickness (= 1.3 μm) but different top width. The one waveguide has a top width of 2.7 μm to satisfy the single-mode condition, and the other has a top width of 10.5 μm to satisfy the multi-mode condition. Supplementary figure 4a shows a transmission spectrum of the multi-mode waveguide coupled to a resonator. As shown in the graph, the transmission spectrum exhibits the Fano resonance. supplementary figure 4b shows a transmission spectrum of the single-mode trapezoidal waveguide coupled to the same resonator. The resonance dips show a well-defined Lorentzian line shape and do not exhibit the Fano resonance. Therefore, it is confirmed that Fano resonance can be readily suppressed by simply controlling the top width of the trapezoidal waveguides.

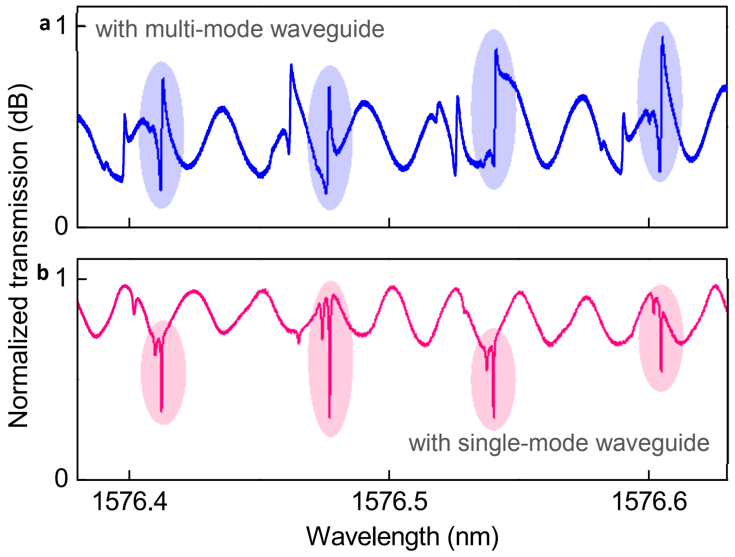

Supplementary Figure 4. Transmission spectra of multi-mode and single-mode waveguides coupled to the same resonator. a Transmission spectrum of the multi-mode waveguide coupled to the resonator. The resonance dips (blue shade) in the graph exhibit Fano line shapes. **b** Transmission spectrum of the single-mode waveguide coupled to the same resonator. The resonance dips (pink shades) in the graph show well-defined Lorentzian line shapes.

Supplementary Note 6. STUDY ON A FLIP-CHIP COUPLING

Supplementary figure 5a describes a system of a waveguide evanescently coupled to a resonator. For a cavity on resonance, an internal field can be described by

$$\frac{da}{dt} = -\frac{1}{2}\left(\sum_i \kappa_i^2 + \kappa_{\text{rad}}^2 + \sigma_0^2 \right) a + i\kappa_0 s \quad (1)$$

where $\kappa_0, \kappa_i, \kappa_{\text{rad}}$ and s are a coupling amplitude to a fundamental mode, a coupling amplitude to an ith higher-order mode, a coupling amplitude to a radiation mode, and a field amplitude of the fundamental mode, respectively. In addition, a and σ_0 are a field amplitude of the resonator and a round-trip amplitude loss coefficient of the resonator, respectively[4].

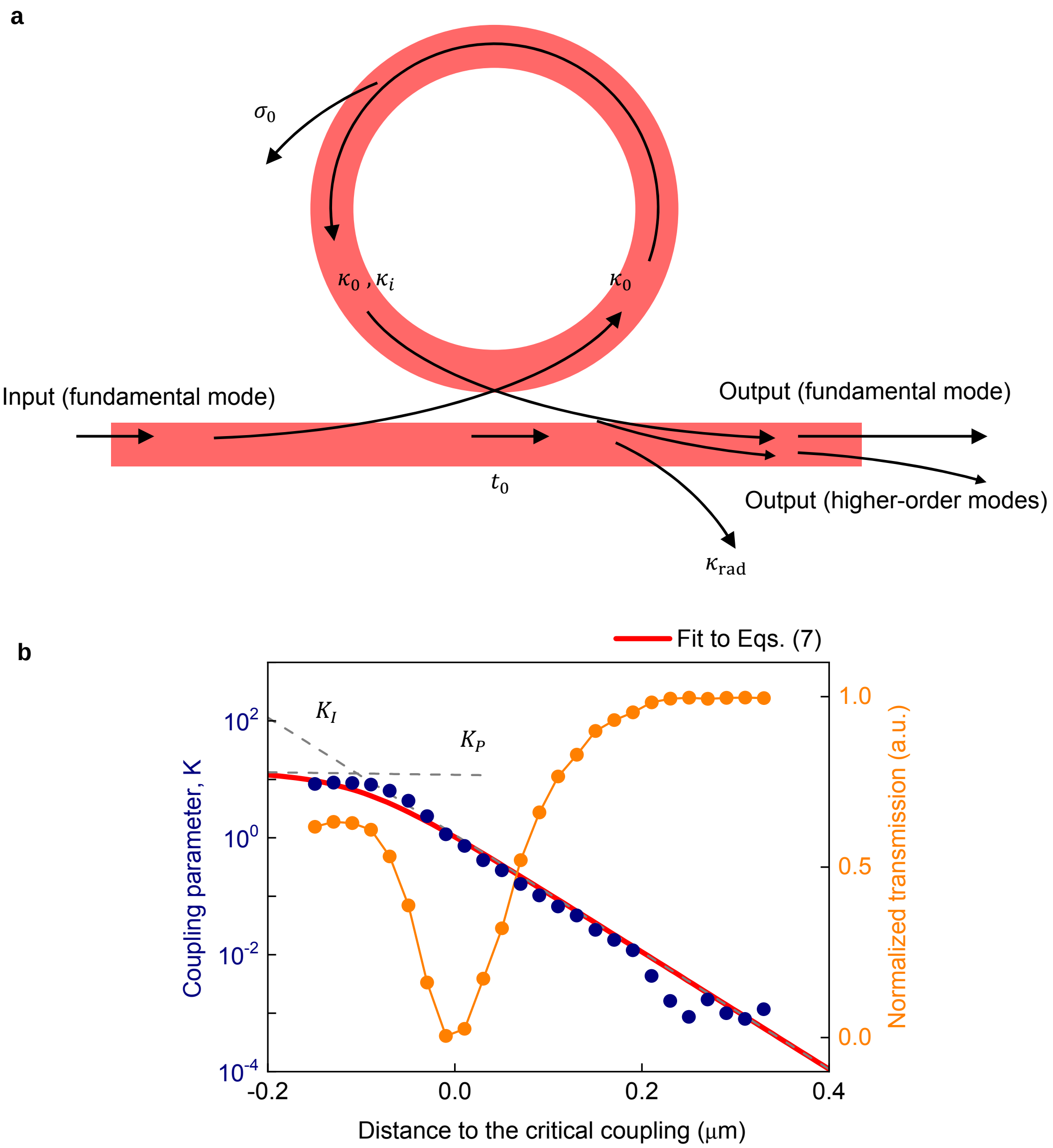

Supplementary Figure 5. Analysis of the coupling of the high-Q resonators and the waveguides. a Schematic of resonator-waveguide coupled system. **b** Measured coupling parameter *K* and normalized transmission *T* versus the gap. K_I and K_P indicate intrinsic and parasitic coupling parameters, respectively.

By solving Eq. (1), the transmission through the waveguide can be expressed by

$$T = \left| t_0 + \frac{i\kappa_0 a}{s} \right|^2 . \quad (2)$$

At a steady-state, the transmission of the waveguide can be expressed as

$$T = \left(\frac{1-K}{1+K} \right)^2 \quad (3)$$

where a coupling parameter K is defined by

$$K \equiv \frac{\kappa_0^2}{\sum_{i\neq 0} \kappa_i^2 + \kappa_{\mathrm{rad}}^2 + \sigma_0^2} \quad (4)$$

K is a ratio of a coupled power to the desired mode (the fundamental mode in our case) to a total power loss of the system. In addition, K can be decomposed into an intrinsic contribution $K_{\mathrm{I}} = \kappa_0^2 / \sigma_0^2$ and a parasitic contribution $K_{\mathrm{P}} = \kappa_0^2 \Big/ \left(\sum_{i\neq 0} \kappa_i^2 + \kappa_{\mathrm{rad}}^2 \right)$, so that $1/K = 1/K_I + 1/K_P$. By inverting Eq. (3), one can calculate K from T using the following equation:

$$K = \frac{1 \pm \sqrt{T}}{1 \mp \sqrt{T}}, \quad (5)$$

where upper signs are for an over-coupled regime and lower signs are for an under-coupled regime. A coupling ideality I is defined as a power coupled to the desired mode divided by a power coupled to all modes and is given by the following equation[4]:

$$I \equiv \frac{\kappa_0^2}{\sum_{i \neq 0} \kappa_0^2 + \kappa_{\text{rad}}^2} = \frac{1}{1 + K_{\text{P}}^{-1}}. \tag{6}$$

For our experiments, the waveguide is designed to have only one mode (the fundamental mode), therefore K_{P} is dominated by the radiation mode. Thus, we can rewrite $K = \kappa_0^2 \big/ \left(\kappa_{\text{rad}}^2 + \sigma_0^2\right)$ and $K_{\text{P}} = \kappa_0^2 \big/ \kappa_{\text{rad}}^2$. Since κ_0^2 and κ_{rad}^2 in the evanescent coupling have an exponential dependence on the gap between the waveguide and the resonator, we can write $\kappa_0^2 = \bar{\kappa}_0^2 \exp\left(-\gamma_0 x\right)$ and $\kappa_{\text{rad}}^2 = \bar{\kappa}_{\text{rad}}^2 \exp\left(-\gamma_{\text{rad}} x\right)$, where γ_0 and γ_{rad} are a spatial decay rate of the fundamental mode and a spatial decay rate of the radiation mode, respectively. Here, $\bar{\kappa}_0^2$ and $\bar{\kappa}_{\text{rad}}^2$ as the proportional constants represent the spatial decay rates at a resonator-waveguide contact (x = 0). Therefore, we can write K as below:

$$K = \frac{\bar{\kappa}_0^2 \exp\left(-\gamma_0 x\right)}{\bar{\kappa}_{\text{rad}}^2 \exp\left(-\gamma_{\text{rad}} x\right) + \sigma_0^2}. \tag{7}$$

By fitting the gap versus K graph using Eq. (7) (supplementary figure 5b, red curve), we can extract κ_0^2, κ_{rad}^2, and σ_0^2. From the extracted values of κ_0^2 and κ_{rad}^2, we can calculate K_{P}, and hence I using Eq. (6). By using this method, we obtain the coupling ideality I of 0.923 at the

critically coupled gap. In general, γ_{rad} is larger than γ_0. As a result, K approaches K_{I} for a large x, and K_{P} for a small x. Therefore, the gap versus K graph (in logarithmic scale) approaches asymptotically to K_{I} for a large x, and to K_{P} for a small x as marked in supplementary figure 5b.

Supplementary Note 7. POLARIZATION DEPENDENCY OF LOSS PERFORMANCE

Here we discuss characteristics of a trapezoidal waveguide according to polarization. As a result of measuring Q-factors of both polarization in the same resonator, the Q-factor of the TM mode is statistically around one-fifth of that of the TE mode at the telecom wavelength. Based on numerical analysis with a volume current method[5,6], we confirmed that the scattering loss of TM mode is 8.90 dB/m while that of TE mode is 1.62 dB/m. This numerical analysis agrees well with the measured result. We attribute this difference to the distribution of their mode profiles. Supplementary figure 6 shows mode profiles for the fundamental TE and TM modes at 1.55 μm wavelength, respectively. While the TE mode is mostly confined at the top of the waveguide, the TM mode stretches to the side slopes of the waveguide. Since the slopes of the waveguide are an etched surface that is rougher than the top surface which is the surface formed by thermal growth, the Q-factor of TM mode is lower than that of TE mode.

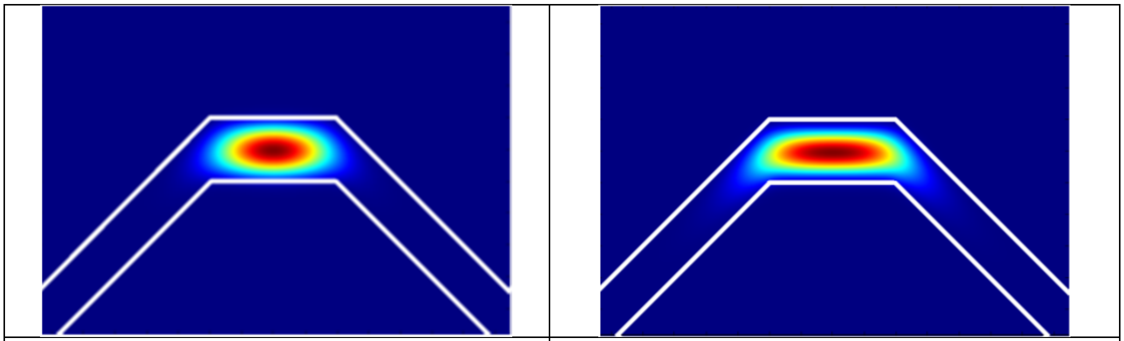

TE mode profile	TM mode profile

Supplementary Figure 6. Comparison of mode profiles of the fundamental TE and TM modes in trapezoidal waveguides.

Supplementary Note 8. DISPERSION ENGINEERING OF TRAPEZOIDAL WAVEGUIDES

The feasibility of dispersion engineering is a crucial element of optical waveguides and resonators for various applications. For example, modulation instability for a positive gain by Kerr nonlinearity only happens under anomalous group velocity dispersion (GVD). On the other hand, normal GVD can be utilized for increasing the coherency of supercontinuum sources. The dispersion properties of the waveguides can be controlled by changing the geometry of the waveguide while the dispersion properties of the materials forming the waveguides are fixed. To confirm the dispersion controllability of the trapezoidal waveguides, we calculated GVD of the trapezoidal waveguides with a finite element method mode solver. Supplementary figure 7a shows the GVD of the trapezoidal waveguide with the various slope angles θ. For the simulation, the core material is selected as As_2S_3 and the material index (with material dispersion considered) is taken from the other literature[7]. The top width w and the thickness of the film d are set to 2 and 1 μm, respectively. As the angle changes from smaller to larger angle, the overall level of the GVD is increased, and a wider range of the wavelength exhibits anomalous dispersion (GVD > 0). In addition, a wavelength where the GVD becomes 0, namely zero-dispersion wavelength (ZDW), is blue-shifted as the angle increases. These trends occur because, with the higher slope angle, the optical mode is more tightly confined in the top core region. The

tighter confinement occurs at the higher slope angle because the thickness difference (= 1-cosθ) between the film on the core and the film on the slope is larger at larger angle θ. As the optical mode is more tightly confined in the top core region, the optical mode experiences more geometric dispersion and deviates from the material dispersion of As_2S_3 (the red dashed line in supplementary figure 7a). Supplementary figure 7b shows the simulated GVD of the trapezoidal waveguide with various top widths w. The slope angle and the thickness of the film are set to 50° and 1 μm, respectively, for the simulations. As the top width changes from 1 to 3 μm, the GVD curve gets flattened over the larger wavelength range and the ZDW red-shifts.

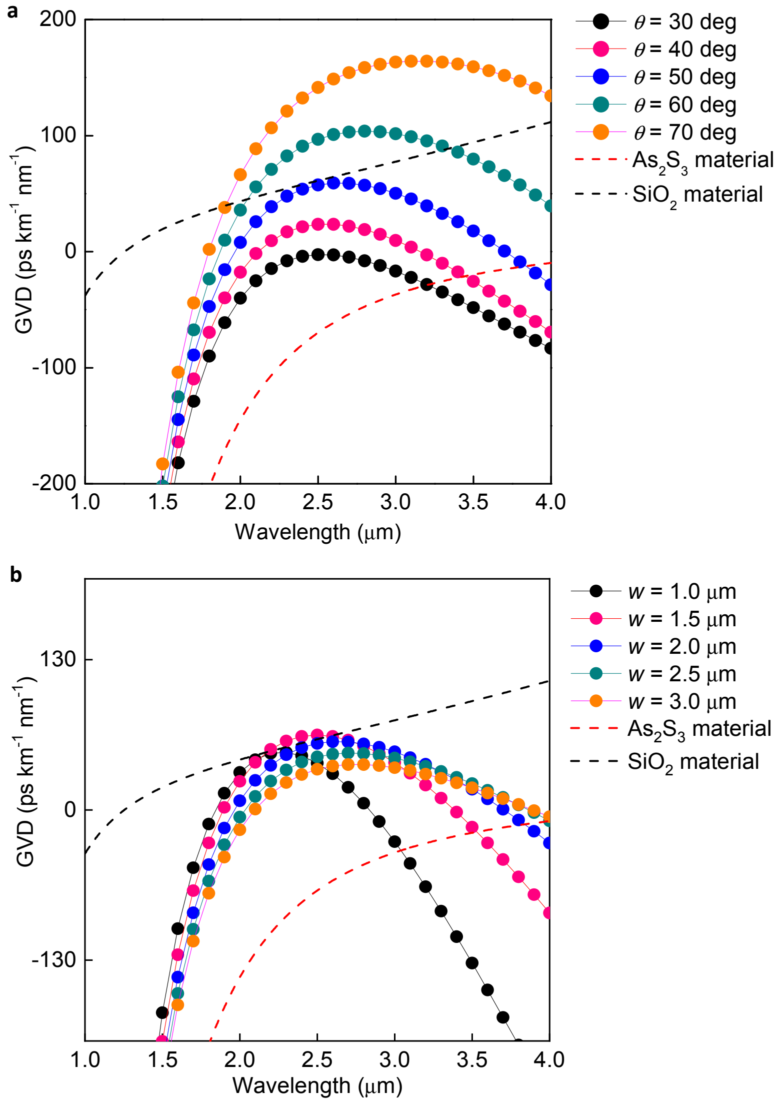
a
b
GVD (ps km-1 nm-1)
Wavelength (µm)
θ = 30 deg
θ = 40 deg
θ = 50 deg
θ = 60 deg
θ = 70 deg
w = 1.0 µm
w = 1.5 µm
w = 2.0 µm
w = 2.5 µm
w = 3.0 µm
As2S3 material
SiO2 material

195

Supplementary Figure 7. GVD spectra of trapezoidal waveguides with various dimensions. **a** GVD spectra of trapezoidal waveguides with different θ while w and t_{core} are fixed to 2 and 1 μm, respectively. **b** GVD spectra with different w. θ and t_{core} are fixed to 50◦ and 1 μm, respectively.

Supplementary Note 9. TRAPEZOIDAL WAVEGUIDE DESIGNS FOR MID-INFRARED APPLICATIONS

Chalcogenide glasses have a very wide transmission window in the mid-infrared (2 to 20 μm) range[8]. Therefore, our trapezoidal waveguides using the chalcogenide films can be used to implement efficient nonlinear optical devices for the mid-infrared. By having a simple modification discussed here, one can readily apply the trapezoidal waveguide structures to the mid-infrared while keeping the high-quality factors and the high optical nonlinearity. The modification is to add a sub-cladding layer[9] under the core layer to isolate the optical mode from the SiO_2 platform structure which may induce material absorption in the mid-infrared. Supplementary figure 8a shows a trapezoidal waveguide design with the suggested modifications. In this design, As_2Se_3 (n = 2.68 at 5 μm wavelength), a high index chalcogenide material, is selected as the core layer. For the sub-cladding layer, As_2S_3 (n = 2.41 at 5 μm wavelength) is chosen since its material index is lower than As_2Se_3. The index values of these materials for the mode simulations are taken from the previous work[7]. Supplementary figure 8b shows the optical mode profile (|κ| field) of the fundamental TE mode of the design at 5 μm wavelength. With the sub-cladding layer, the optical fields are well isolated from the SiO_2

platform structure. The absorption loss by the SiO_2 platform is calculated using Lumerical Mode Solutions with the refractive index and the extinction coefficient (n = 1.34, κ = 0.006 at 5 μm wavelength) taken from the other work[10]. The calculated absorption loss due to SiO_2 absorption is only 5.97 dB/km. In terms of quality factors, this propagation loss is equivalent to the quality factor of $2.41 \cdot 10^{11}$. Thus, one can neglect the loss due to the SiO_2 absorption in this sub-cladded structure. Besides, in terms of the dispersion controllability, the multi-cladding layer gives additional degrees of freedom as compared to the single-layer structure.

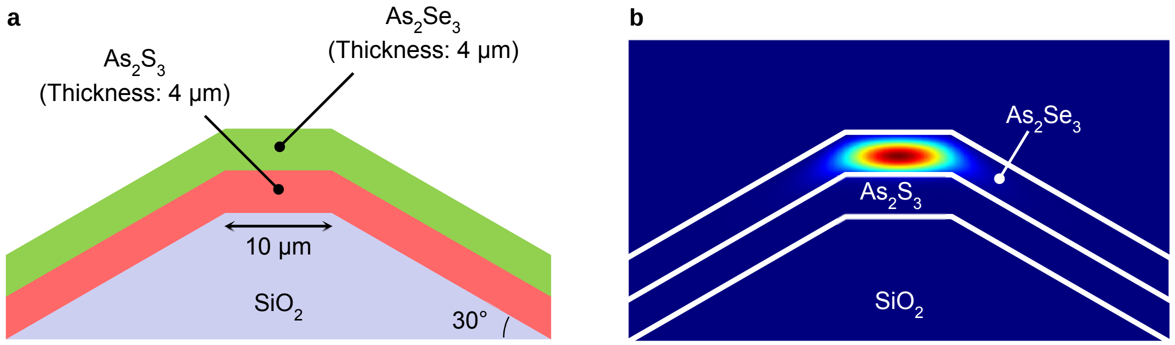

Supplementary Figure 8. An example of sub-cladded trapezoidal waveguide designs. a Schematic of the sub-cladded trapezoidal waveguide structure. **b** Simulated $|E|$ field profile of fundamental mode of the waveguide. The optical fields are isolated from the SiO_2 platform structure by the 4 μm-thick sub-cladding (As_2S_3) layer.

Supplementary Note 10. PROCESS COMPATIBILITY

We confirm that trapezoidal waveguides can be realized through various deposition techniques regardless of their deposition directionality. In principle, trapezoidal waveguides should have a thickness contrast between the top flat and the wedge areas to confine light. However, not all

deposition method has the directionality required for the thickness contrast. For example, chemical vapor deposition (CVD) is of high conformality contrary to PVD. Moreover, CVD is a widely used deposition method due to its remarkable utility for various precursors, reactive organic and inorganic materials as well as inert materials.

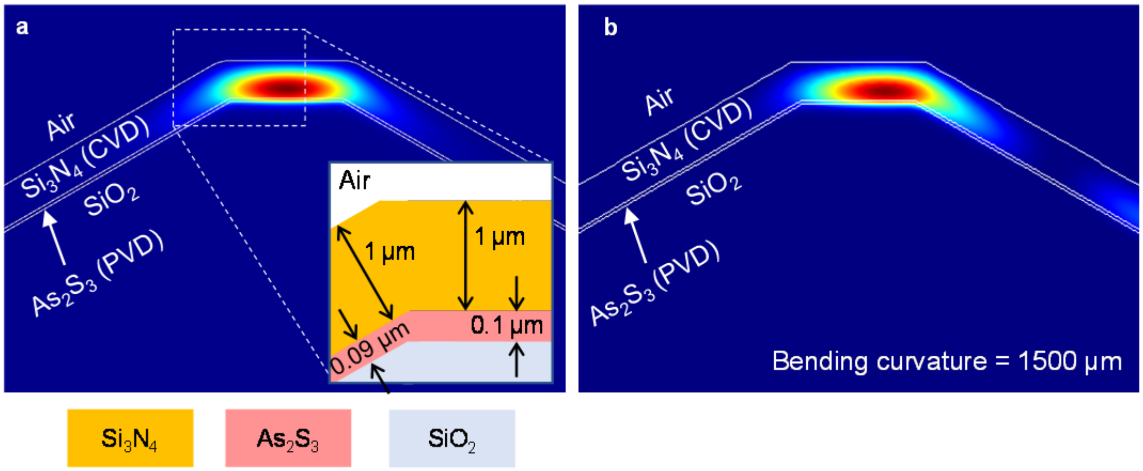

Supplementary Figure 9. Mode confinement of trapezoidal waveguides with a core deposited through CVD. a The simulated $|E|^2$ profiles of a trapezoidal waveguide having a conformal Si_3N_4 film as a core layer. The bottom clad of a 0.1 μm As_2S_3 is introduced at the bottom of the core to make a thickness contrast (inset). **b** The mode profile of the fundamental TE mode for the ring resonator with a radius of 1500 μm. In the both simulations, the top width and the wedge angle are 3 are 3 μm and 30°, respectively.

As shown in supplementary figure 9a, a simple idea introducing the sub-cladding can be used to confine lights laterally in a conformal layer. It is assumed that a trapezoidal waveguide is composed of two layers: the one is a 0.1 μm thick As_2S_3 (n~2.43) layer deposited by an ideally directional deposition (or PVD), and the other is a 1 μm thick Si_3N_4 (n~2.00) layer deposited by

an ideally conformal deposition (or CVD). Supplementary figure 9a displays the corresponding intensity profile of the fundamental TE mode at 1.55 μm wavelength. By the aid of the thin PVD film with a thickness contrast of 0.866 (= cos30°), the optical modes can be confined to the top flat region even though the CVD film has no thickness contrast. The 86.4% and 7.7% of optical powers are confined inside the CVD and PVD films, respectively. Since the thickness difference which can be induced by the thin sublayer is quite small compared to the total thickness of the deposited films, we numerically evaluated the robustness of this approach to bending, practically the most important perturbation. As shown in supplementary figure 9b, for the bent waveguide with a curvature of 1500 μm (smaller than the radius used for SBS experiments in this paper), it is confirmed that 86.8% of the optical power is confined in the core and bending loss is ignorable. This simulation result supports that the proposed light-guiding structure can be fabricated even with conformal deposition techniques by introducing a thin sub-layer that is directionally deposited.

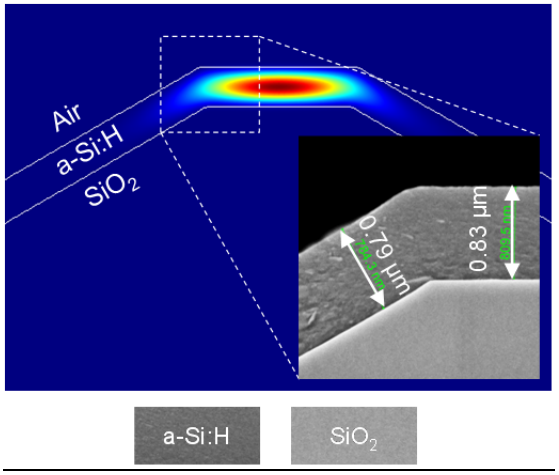

Supplementary Figure 10. Mode confinement of trapezoidal waveguides with a core deposited through PECVD. The simulated $|E|^2$ profiles of the fabricated trapezoidal waveguide

having an a-Si film as a core layer. The normal thickness of the core layer deposited by PECVD is 0.83 μm and 0.73 μm on the top and the slope, respectively (inset). The top width and the wedge angle are 3 μm and 30°, respectively.

In a separate experiment, as shown in the inset of supplementary figure 10, we deposited an hydrogenerated amorphous Si film of 830 nm thickness on a trapezoidal structure using a plasma-enhanced chemical vapor deposition (PECVD) technique to check the thickness contrast. Although the deposition was performed by a conventional recipe without any exceptional modifications to attain directionality, the measured thickness contrast is 0.95 which is considerably lower than 1, standing for the case of ideal conformal deposition. Supplementary figure 10 displays the corresponding intensity profile of the fundamental TE mode at 1.55 μm wavelength. Considering the versatility of PECVD and its practical deviation from ideal conformality we observed, it is inferred that the proposed trapezoidal waveguides can be implemented with a variety of materials."

Supplementary Note 11. STRATEGY TO EMPLOY MATERIALS HAVING A LOWER REFRACTIVE INDEX AS A CORE

We suggest an example in which material having a lower refractive index than silica is employed as a waveguide core, by introducing a sub-cladding structure that has a lower index than the material to be used as a core as shown in supplementary figure 11.

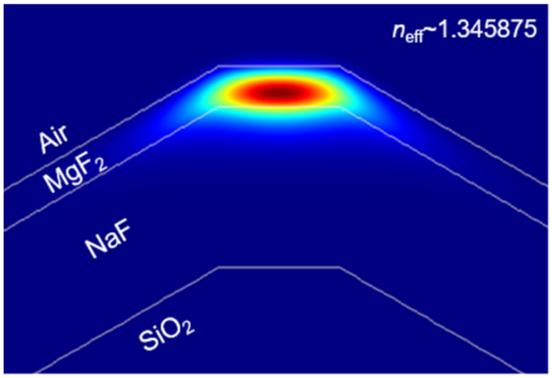

Supplementary Figure 11. Bypass strategy to make sub-structures with other materials rather than silica. The simulated $|E|^2$ profiles of an MgF_2 trapezoidal waveguide with a sub-structure of NaF-on-SiO_2.

It is supposed that the waveguide consists of a 1 μm thick MgF_2 (n~1.4172) core and a 4 μm thick NaF (n~1.3194) sub-cladding layer which are successively deposited on a SiO_2 (n~1.4657) bottom platform. The COMSOL simulation shows that the TE mode profile is well confined inside the MgF2 core area which is optically separated from the silica platform structure. Here, 67.5 % of optical power is confined in the core, and the mode overlap with the bottom silica platform is suppressed to $4.303 \cdot 10^{-6}$.

Supplementary References

1. H. A. Haus, *Waves and fields in optoelectronics*. (Prentice-Hall, 1984).
2. Saitoh, T., Mukai, T. & Mikami, O. Theoretical analysis and fabrication of antireflection coatings on laser-diode facets. *Journal of Lightwave Technology* **3**, 288-293 (1985).

3. Chiba, A., Fujiwara, H., Hotta, J.-i., Takeuchi, S. & Sasaki, K. Fano resonance in a multimode tapered fiber coupled with a microspherical cavity. *Applied Physics Letters* **86**, 261106 (2005).
4. Spillane, S., Kippenberg, T., Painter, O. & Vahala, K. Ideality in a fiber-taper-coupled microresonator system for application to cavity quantum electrodynamics. *Physical review letters* **91**, 043902 (2003).
5. Johnson, S.G., Povinelli, M.L., Soljačić, M., Karalis, A., Jacobs, S. & Joannopoulos, J.D. Roughness losses and volume-current methods in photonic-crystal waveguides. *Applied Physics B* **81**(2-3), 283-293 (2005).
6. Kita, D.M., Michon, J., Johnson, S.G. & Hu, J. Are slot and sub-wavelength grating waveguides better than strip waveguides for sensing? *Optica* **5**(9), 1046-1054 (2018).
7. Zhang, L., Agarwal, A. M., Kimerling, L. C. & Michel, J. Nonlinear Group IV photonics based on silicon and germanium: from near-infrared to mid-infrared. *Nanophotonics* **3**, 247-268 (2014).
8. Adam, J.-L. & Zhang, X. *Chalcogenide glasses: preparation, properties and applications*. (Woodhead publishing, 2014).
9. Yu, Y., Gai, X., Ma, P., Vu, K., Yang, Z., Wang, R., Choi, D.-Y., Madden, S. & Luther-Davies, B., Experimental demonstration of linearly polarized 2–10 μm supercontinuum generation in a chalcogenide rib waveguide. *Optics letters* **41**, 958-961 (2016).
10. Kitamura, R., Pilon, L. & Jonasz M., Optical constants of silica glass from extreme ultraviolet to far infrared at near room temperature. *Applied optics* **46**, 8118-8133 (2007).
